# Malignant fibrous histiocytoma amplified sequence 1 alleviates inflammation and renal fibrosis in diabetic nephropathy by inhibiting TLR4

**DOI:** 10.1042/BSR20190617

**Published:** 2019-11-12

**Authors:** Huan Lian, Yi Cheng, Xiaoyan Wu

**Affiliations:** 1 Department of Nephrology, Zhongnan Hospital of Wuhan University, Wuhan 430071, P.R. China; 2 Tongji Hospital, Tongji Medical College of Huazhong University of Science and Technology, Wuhan 430030, P.R. China

**Keywords:** animal model, Diabetic nephropathy, MFHAS1, renal fibrosis, TLR4

## Abstract

**Background:** Diabetic nephropathy (DN) is the most common complication of diabetes mellitus (DM). The signal pathway and molecular mechanism of renal fibrosis are not fully understood. In the present study, we aimed to explore the function of malignant fibrous histiocytoma amplified sequence 1 (MFHAS1) in DN.

**Method:** Mouse mesangial cells (MMCs) were treated with low glucose (LG) or high glucose (HG). TAK242 or short hairpin TLR4 (shTLR4) were employed to down-regulate Toll-like receptor 4 (TLR4). The effect of MFHAS1 knockdown or overexpression on fibrosis-related factors, inflammatory factors and TLR4 in MMCs were examined after transfecting with short hairpin RNA (shRNA) or MFHAS1 overexpressed plasmid, respectively. The expression levels of MFHAS1, inflammatory factors, fibrosis factors and TLR4 in db/db or streptozotocin (STZ) mice tissues and MMCs were examined by quantitative real-time polymerase chain reaction (qRT-PCR) and Western blot. The effect of MFHAS1 overexpression *in vivo* was also evaluated.

**Results:** The expression of MFHAS1 in db/db or STZ mice and HG-treated MMCs were significantly increased compared with normal control mice and LG-treated MMCs. Overexpression of MFHAS1 inhibited the expression of inflammatory and fibrotic factors, while knockdown of MFHAS1 promoted them. MFHAS1 suppressed the activation of TLR4 pathway via inhibiting the expression of TLR4, and then alleviating inflammation and fibrosis in DN. MFHAS1 overexpression *in vivo* improved the symptoms of STZ-induced DN mice.

**Conclusion:** The current study demonstrated that MFHAS1 relieved inflammation and renal fibrosis in DN mice via inhibiting TLR4. The results revealed that the MFHAS1 may be a molecular target in DN therapy.

## Introduction

Diabetic nephropathy (DN) is the most common cause of chronic kidney disease (CKD) [1]. DN, one of the most important complications of diabetes mellitus (DM), is the primary cause of end-stage renal dialysis (ESRD) in Western countries [2]. Globally, patients with DN account for approximately 30–50% of patients requiring renal replacement therapy [3]. According to the Chinese National Dialysis Registration quality control analysis data in 2012, 18.4% of new hemodialysis patients suffer from DN and 17.5% of new peritoneal dialysis patients suffer from DN, which is the second cause of ESRD and dialysis in China [4].

DN is clinically characterized by persistent proteinuria, decreased renal function and elevated blood pressure [5]. Pathologically, it is characterized by proliferation of mesangium and mesangial matrix [6], formation of K-W nodules [7], vitreous changes in the entry and exit arterioles [8]. Pathological changes of DN mainly include glomerular sclerosis (GS) and renal tubular interstitial fibrosis (RTIF) [9]. Many reports suggested that glomerular injury played a dominant role in the pathogenesis of DN [10]. Mesangial cells are an important part of glomerular intrinsic cells, and their number, morphology and location are relatively stable under normal circumstances, playing an important role in maintaining the normal structure and function of the kidney [11,12]. In recent years, the apoptosis of mesangial cells has attracted more and more attention as a potential contributor to renal lesions [13]. Mesenchymal cell apoptosis can be induced by long-term high glucose (HG) in the blood medium [14]. The loss of mesangial cells is the main pathological mechanism of kidney injury caused by DM [15].

Malignant fibrous histiocytoma amplified sequence 1 (MFHAS1), a member of the C-terminal of Ras of complex proteins (ROCO) protein family [16,17], was initially found in malignant fibrous histiocytoma, it could regulate TLR-dependent signaling pathway, which was closely related to the occurrence and development of tumors [18]. Previous studies on MFHAS1 were mostly reported in cancer-related and inflammation-related researches. Chen et al. [19] reported that MFHAS1 promoted the progress of colorectal cancer through modulating polarization of cancer-associated macrophages by STAT6 signaling pathway. Wang et al. [20] found that overexpression of MFHAS1, stimulated by HG, could relieve inflammation by Akt/HO-1 signaling Pathway in Human Umbilical Vein Endothelial Cells. Toll-like receptor 4 (TLR4), a recognition receptor, activates downstream signal transduction pathway to release inflammatory factors to resist bacterial invasion by recognizing lipopolysaccharide (LPS) components in cell wall of Gram-negative bacteria. It has been found that the alteration of TLR4 expression in both human and animal can lead to abnormal response of effector cells to LPS. Shi et al*.* [21] reported that MFHAS1 inhibited TLR4 signaling pathway via induction of suppression of c-Jun dephosphorylation at Thr^239^ and protein phosphatase 2A (PP2A) C subunit cytoplasm translocation. Moreover, previous study indicated that MFHAS1 was associated with mesenchymal stem cells and fibroblasts in mouse lung telocytes [22]. However, the functions of MFHAS1 on inflammation and renal fibrosis in DN are not understood.

In the current study, we found that the expression of MFHAS1 increased in db/db and streptozotocin (STZ)-induced DN mice in order to alleviate DN symptoms. Up-regulation of MFHAS1 could inhibit the expression of inflammatory and fibrotic factors by inhibiting the activation of TLR4 pathway. In addition, we also found that overexpression of MFHAS1 *in vivo* could improve the symptoms of STZ-induced DN mice. These results provided new molecular therapies and strategies for the treatment of DN.

## Materials and methods

### Cell culture

Mouse mesangial cells (MMCs) were obtained from Cell Bank Type Culture Collection of the Chinese Academy of Sciences (Shanghai, China). Cells were cultured in DMEM (Corning Life Sciences, New York, U.S.A.) supplemented with 10% fetal bovine serum (FBS) (Invitrogen, Shanghai, China), 100 μg/ml streptomycin (Corning Life Sciences) and 100 units/ml penicillin (Corning Life Sciences), and incubated in a humidified atmosphere at 37°C with 5% CO_2_. Cells were treated with low glucose (LG, 5.5 mM) and HG (20 mM) for 24 h.

### Cell transfecion and reagents

Overexpression of MFHAS1 was achieved using pcDNA MFHAS1 plasmid (GenePharma Co. Ltd., Shanghai, China) transfection, with an empty vector as a control. Short hairpin RNA (shRNA), short hairpin TLR (shTLR) and short hairpin negative control (shNC) RNAs were synthesized and provided by GenePharma. MMCs were cultured to 60–70% confluence and then transfected with Lipofectamine 2000 Reagent (Invitrogen) according to the manufacturer’s instructions. After transfection, the expression level of MFHAS1 and TLR4 was validated by quantitative real-time polymerase chain reaction (qRT-PCR).

### RNA extraction and qRT-PCR

Total RNA was extracted from the mice renal tissues and MMCs using TRIzol (Invitrogen Carlsbad, CA, U.S.A.) and treated with DNase I (Roche, Indianapolis, IN, U.S.A.) to remove residual DNA according to the manufacturer’s protocol. qRT-PCR was used to examine the expression levels of MFHAS1, inflammatory factors (TNF-α, IL-1β and IL-6), fibrosis-related factors (TGF-β, fibronectin and collagen IV) and TLR4. A total of 2 μg RNA was reverse transcribed to cDNA with oligo (dT) primers using a cDNA synthesis kit (TaKaRa Biotechnology Co., Ltd, Dalian, China), and then used for quantitative PCR with SYBR Green qPCR Master Mixes (Molecular Probes, Invitrogen) according to the manufacturer’s instructions. β-actin mRNA levels were used for normalization. The oligonucleotides used as PCR primers were as follows: MFHAS1 5′-GGAGAGAGTGGAGGGATGC-3′ and 5′-GGAGGTGACGGTGGGTAG-3′, TNF-α 5′-CCCTCCTTCAGACACCCT-3′ and 5′-GGTTGCCAGCACTTCACT-3′, IL-1β 5′-TTGAGTCTGCCCAGTTCC-3′ and 5′-TTTCTGCTTGAGAGGTGCT-3′, IL-6 5′-CAATAACCACCCCTGACC-3′ and 5′-GCGCAGAATGAGATGAGTT-3′, TGF-β 5′-ACCACACCAGCCCTGTTC-3′ and 5′-CGTCAGCACCAGTAGCCA-3′, fibronectin 5′- ATTCTGTAGGCCGTTGGA-3′ and 5′-TACTGCTGGATGCTGATGA-3′, collagen IV 5′-ATGCCCTTTCTCTTCTGCAA-3′ and 5′-GAAGGAATAGCCGATCCACA-3′, β-actin 5′-GCTCGTCGTCGACAACGGCTC-3′ and 5′-CAAACATGATCTGGGTCATCTTCTC-3′; the ABI 7300 system (Applied Biosystem, Foster City, CA, U.S.A.) was programmed to initially incubate the samples at 95°C for 10 min, and then at 95°C for 10 min, followed by 40 cycles of incubation at 95°C for 15 s and 60°C for 45 s. Fold changes were calculated using the 2^−ΔΔ*C*_t_^ method. All data represent the average of three replicates.

### Western blot

Protein lysates were extracted using 500 μl radioimmunoprecipitation assay (RIPA) buffer with 1 mM phenylmethane sulfonyl fluoride (Sigma, St. Louis, MO, U.S.A.). After blocking in Tris-buffered saline containing 0.1% Tween-20 (TBS-T) with 5% nonfat dry milk for 30 min at 37°C, membranes were washed four times in TBS-T and incubated with primary antibodies overnight at 4°C. Primary antibodies were all obtained from Abcam (Cambridge, MA, U.S.A.) and used at the following dilutions: anti-β-actin (1:500; ab8226), anti-MFHAS1 (1:250; ab185289), anti-TNF-α (1:1000; ab6671), anti-IL-1β (1:1000; ab9722), anti-IL-6 (1:20; ab7737), anti-TGF-β (1:1000; ab170874), anti-fibronectin (1:1000, ab45688) and anti-collagen IV (1:1000, ab6586). Following extensive washing, membranes were incubated with horseradish peroxidase-linked goat polyclonal anti-rabbit IgG secondary antibody (cat. no. 7074; CST Biological Reagents Co., Ltd., Shanghai, China) at a dilution of 1:2000 for 1 h at room temperature. β-actin served as the loading control.

### Experimental animals

The animal study protocol was approved by the Institutional Animal Care and Use Committee of Zhongnan Hospital of Wuhan University. Male C57BL/6 mice (*n*=6/group), pathogen-free male db/m mice (non-diabetic animal model, *n*=6/group), and db/db mice (type 2 DM animal model, *n*=6/group) were purchased from Beijing HFK Bioscience Co., Ltd. (Beijing, China), and housed in barrier facilities with rodent chow and water under a specific pathogen-free (SPF) condition. We developed a DN model by combining STZ in C57BL/6 mice. Mice were intraperitoneally injected with STZ (STZ dissolved in 0.01 mol/l sterile citric acid-sodium citrate buffer at pH 4.5) at dose (55 mg/kg) for 5 consecutive days, and fasting blood glucose was measured after the last injection for 72 h. The STZ mice model were successfully established with blood sugar (>16.7 mmol/l) and urine sugar positive. It is an accelerated type 2 DN model (<4 weeks). First, the kidneys of pathogen-free db/m mice (control group), STZ mice and db/db mice were collected to detect the expression of MFHAS1 after killing. Then, the other STZ groups were injected every second day via the tail vein with the control plasmid (CT) or MFHAS1 overexpression plasmid at a dose of 10 mg/kg/day for 4 weeks. In this step, the mice were divided into four groups: Control group, STZ group, STZ + CT group and STZ + MFHAS1 group. At the 12th week, we collected and harvested the kidneys. The kidneys were fixed in 4% paraformaldehyde for further testing. The expression levels of MFHAS1, inflammatory factors and fibrosis-related factors were measured via qRT-PCR and western blot.

### Statistical analysis

Data were expressed as mean ± standard deviation (SD). Statistical analysis was performed using SPSS 20.0 software (SPSS, Chicago, IL, U.S.A.). Two-tailed Student’s *t* test was performed to compare the differences between two groups and one-way analysis of variance (ANOVA) followed by Dunnett’s multiple comparison was applied to compare the differences among three independent groups. *P*<0.01 was considered statistically significant.

## Results

### MFHAS1 expression level increased in db/db or STZ-induced DN mice and HG induced MMCs

**Figure 1:**
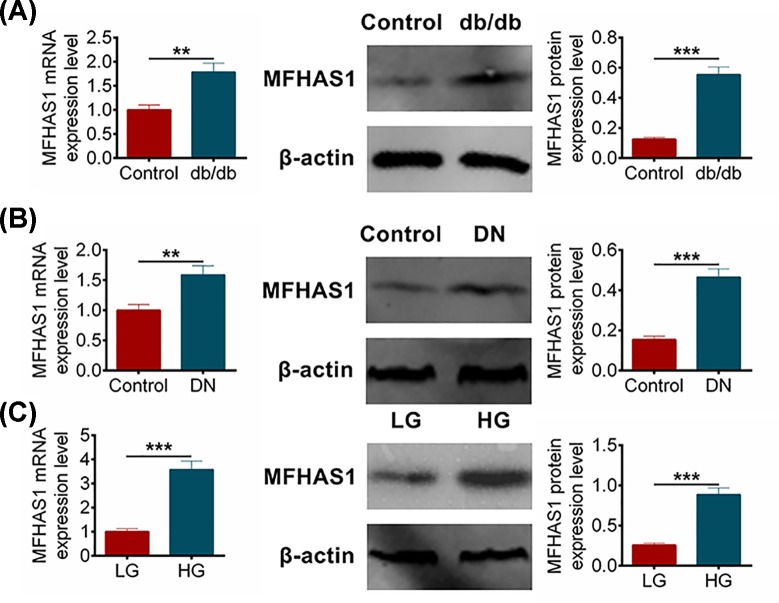
MFHAS1 expression increased in DN tissues and cells (**A**) Relative expression levels of MFHAS1 in db/db mice tissues and normal tissues were identified using qRT-PCR and Western blot. (**B**) Relative expression levels of MFHAS1 in STZ mice tissues and normal tissues were identified using qRT-PCR and Western blot. (**C**) Relative expression levels of MFHAS1 in MMCs under LG treatment and HG treatment identified using qRT-PCR and Western blot. Data were shown as mean ± SD. **P<0.01, ***P<0.001 compared with Control group or LG group.

In the beginning of the study, the expression levels of MFHAS1 in db/db mice, STZ mice and MMCs were detected by qRT-PCR and Western blot. The expression level of MFHAS1 in db/db mice was 1.785 ± 0.185 or 0.554 ± 0.052 measured by qRT-PCR or Western blot, respectively, while the expression level in control group was 1 ± 0.105 (qRT-PCR) or 0.125 ± 0.014 (Western blot) (Figure 1A). Figure 1B showed that the expression levels of MFHAS1 were 1.587 ± 0.152 (qRT-PCR) or 0.465 ± 0.041 (Western blot) in STZ mice and 1 ± 0.098 (qRT-PCR) or 0.154 ± 0.017 in control group. These data exhibited that MFHAS1 was significantly up-regulated in DN tissues compared with normal tissues (*P*<0.001). In addition, we respectively treated MMCs with LG or HG, and then examined the MFHAS1 expression. Figure 1C revealed that the expression level of HG MMCs was 3.578 ± 0.356 (qRT-PCR) or 0.886 ± 0.084 while the expression level of LG MMCs was 1 ± 0.121 (qRT-PCR) or 0.257 ± 0.026 (Western blot). Obviously, the expression level of MFHAS1 in HG MMCs was increased compared with LG MMCs (*P*<0.001). These results indicated that MFHAS1 was markedly up-regulated in both DN tissues and cells compared with that in normal tissues and cells (*P*<0.001).

### MFHAS1 affected the level of DN cellular inflammation and fibrosis

**Figure 2:**
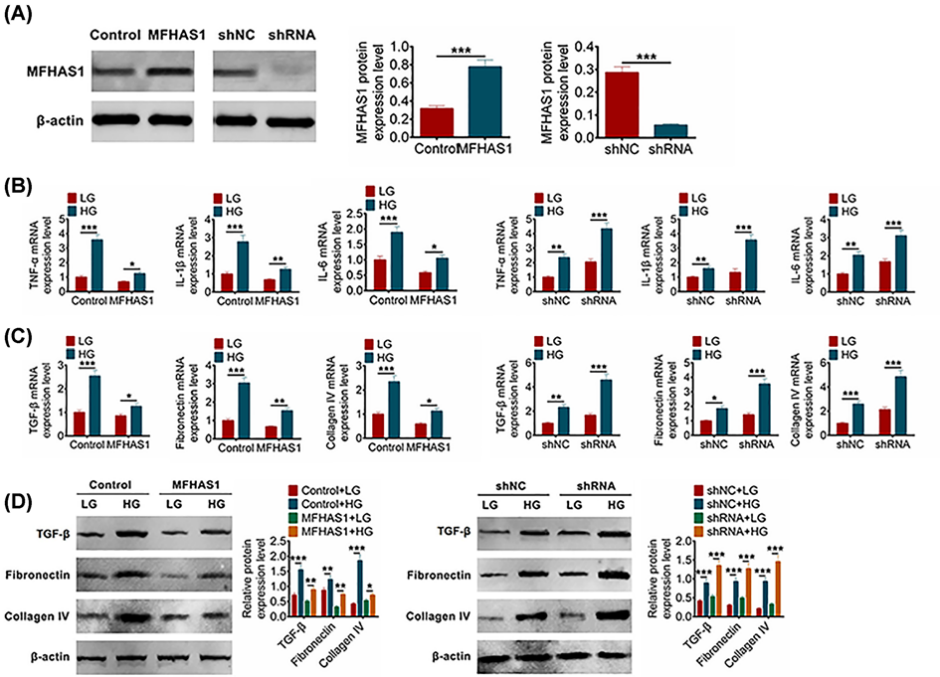
Overexpression of MFHAS1 inhibited inflammatory and fibrosis in MMCs (**A**) MFHAS1 expression levels under LG or HG treatment were detected using Western blot after transfection with MFHAS1 overexpression plasmid, shRNA or shNC in MMCs. (**B**) TNF-α, IL-1β and IL-6 expression levels under LG or HG treatment were detected using qRT-PCR after transfection with MFHAS1 overexpression plasmid, shRNA or shNC in MMCs. (**C**) TGF-β, fibronectin and collagen IV expression levels under LG or HG were detected using qRT-PCR after transfection with MFHAS1 overexpression plasmid, shRNA or shNC in MMCs. (**D**) The expression levels of TGF-β, fibronectin and collagen IV under LG or HG treatments were detected using Western blot after transfection with MFHAS1 overexpression plasmid, shRNA or shNC in MMCs. Data were shown as mean ± SD. *P<0.05, **P<0.01, ***P<0.001 compared with control, shNC, control + LG, MFHAS1 + LG, shNC + LG or shRNA + LG groups.

In order to explore the effects of MFHAS1 on DN cells, the overexpression of MFHAS1 plasmid, control plasmid, shRNA and shNC were constructed and then respectively trasfected into MMCs. The transfection efficiency was examined via Western blot. Figure 2A showed that MFHAS1 was successfully up-regulated in MFHAS1 group and down-regulated in shRNA group. After transfection, MMCs were treated with LG or HG. Primarily, the expression of inflammatory factors and fibrosis-related factors of the four groups were all obviously higher in HG MMCs than that in LG MMCs (Figure 2B-D). The expression levels of inflammatory factors (TNF-α, IL-1β and IL-6) were subsequently measured by qRT-PCR. TNF-α, IL-1β and IL-6 were all significantly inhibited in MFHAS1 group compared with control group (*P*<0.001) while they were dramatically increased in shRNA group compared with shNC group (*P*<0.001) (Figure 2B). In addition, the expression levels of fibrosis-related factors (TGF-β, fibronectin, collagen IV) were detected via qRT-PCR and Western blot, too. Figure 2C,D showed that TGF-β, fibronectin and collagen IV expressions were all obviously suppressed in MFHAS1 group when compared with control group (*P*<0.001), and they were singly enhanced after shRNA trancfection when compared with shNC group (*P*<0.001). Above results disclosed that MFHAS1 up-regulation inhibited the DN cellular inflammation and fibrosis while MFHAS1 knockdown promotes them.

### MFHAS1 inhibited the activation of the bTLR4 pathway via suppressing the expression level of TLR4

**Figure 3:**
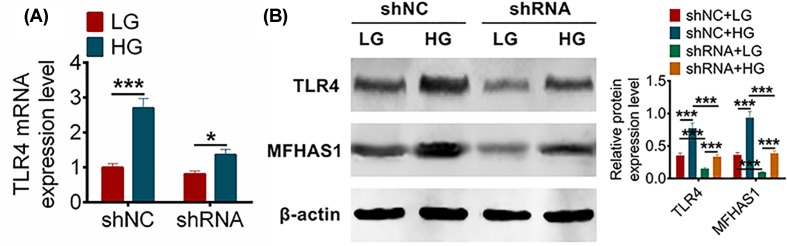
MFHAS1 inhibited fibrosis by inhibiting TLR4 (**A**) TLR4 expression levels under LG or HG were detected using qRT-PCR after transfection with shRNA or shNC in MMCs. (**B**) TLR4 and MFHAS1 expression levels under LG or HG were detected using western blot after transfection with shRNA or shNC in MMCs. Data were shown as mean ± SD. *P<0.05, ***P<0.001 compared with shNC, shNC + LG or shRNA + LG groups.

In the last section, we effectively transfected the shRNA and shNC into MMCs and subsequently induced the MMCs with LG or HG treatment. In order to explore the signaling pathway of MFHAS effecting DN, we examined the expression level of TLR4 by qRT-PCR and Western blot. Whether shNC group or shRNA group, the expression of TLR4 under HG treatment was significantly higher than that under LG treatment (Figure 3A,B). The result of qRT-PCR showed that the expressions of TLR4 under HG treatment were 2.700 ± 0.274 and 1.365 ± 0.154 in shRNA group and shNC group, respectively (Figure 3A). And the result of Western blot under HG treatment were 0.976 ± 0.087 and 0.536 ± 0.053 in shRNA group and shNC group, respectively (Figure 3B). According to these results of qRT-PCR and Western blot, the expression level of TLR4 of shRNA group was obviously inhibited when compared with shNC group under HG treatment (*P*<0.001). At the same time, the expression level of MFHAS1 of shRNA group was significantly increased compared with shNC group (*P*<0.001) (Figure 3B). These results revealed that MFHAS1 inhibited the activation of TLR4 pathway by inhibiting the expression of TLR4.

### MFHAS1 inhibited inflammation and fibrosis by inhibiting activation of TLR4 pathway

**Figure 4:**
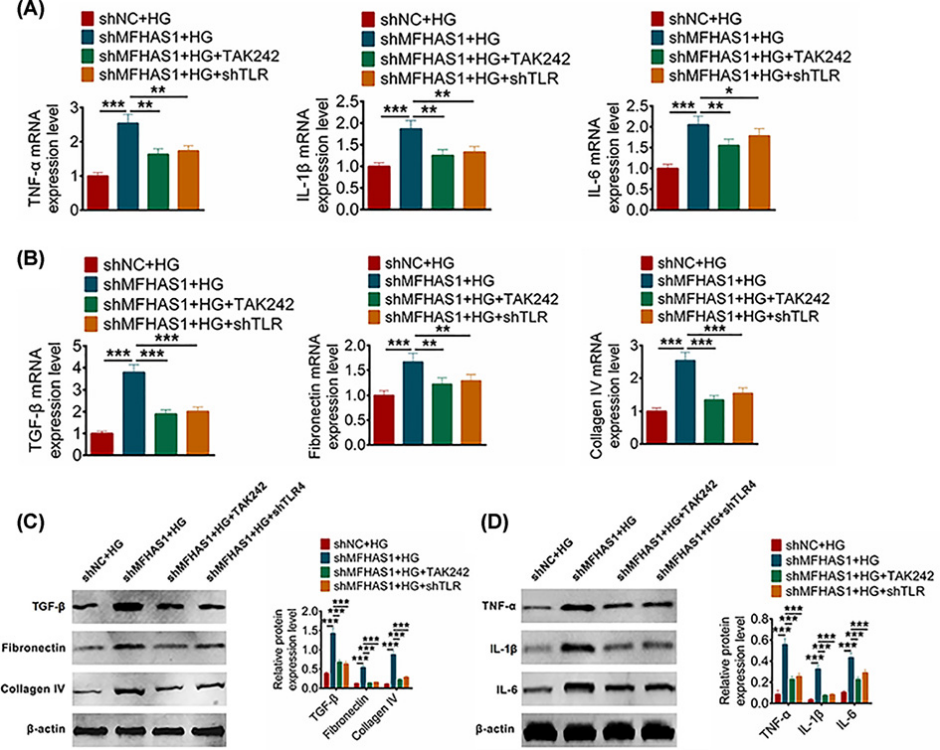
MFHAS1 inhibited inflammation and fibrosis by inhibiting TLR4 pathway MMCs under HG were divided into four groups with shNC, shMFHAS1, shMHAS1 + shTLR4 or shMFHAS1 + TAK242 transfection. (**A**) TNF-α, IL-1β and IL-6 expression levels under HG were detected using qRT-PCR after transfection. (**B**) TGF-β, fibronectin and collagen IV under HG expression levels were detected using qRT-PCR after transfection. (**C**) GFb, fibronectin and collagen IV under HG expression levels were detected using Western blot after transfection. (**D**) TGF-β, fibronectin and collagen IV under HG expression levels were detected using Western blot. Data were shown as mean ± SD. **P<0.01, ***P<0.001 compared with shNC + HG group.

In the previous section, we elaborated that MFHAS1 could inhibit the expression of TLR4. To further verifying the molecular mechanism of MFHAS1 effecting inflammation and fibrosis in DN, shTRL4 TAK242 was used to knockdown TLR4, and then the shMFHAS1 was co-transfected with shTRL4 or TAK242. MMCs were divided into four groups: shNC, shMFHAS1, shMFHAS1 + shTRL4 and shMFHAS1 + TLR4, all of these groups were treated with HG. And then the expression levels of inflammatory factors (TNF-α, IL-1β and IL-6) and fibrosis-related factors (TGF-β, fibronectin and collagen IV) were examined via qRT-PCR and Western blot. Data from qRT-PCR and Western blot showed that TNF-α, IL-1β and IL-6 expressions were all decreased with TLR4 knockdown when the MFHAS1 was down-regulated (Figure 4A,D). The expression levels of TGF-β, fibronectin and collagen IV were significantly inhibited in shMFHAS1 + shTRL4 group and shMFHAS1 + TAK242 group compared with shMFHAS1 group (*P*<0.01) (Figure 4B,C). These results showed that TLR4 knockdown inhibited inflammatory factors and fibrosis-related factors when the MFHAS1 was down-regulated. It indicated that MFHAS1 suppressed inflammation and fibrosis through inhibiting the activation of TLR4 signaling pathway.

### Overexpression of MFHAS1 *in vivo* improved the symptoms of STZ-induced DN mice

**Figure 5:**
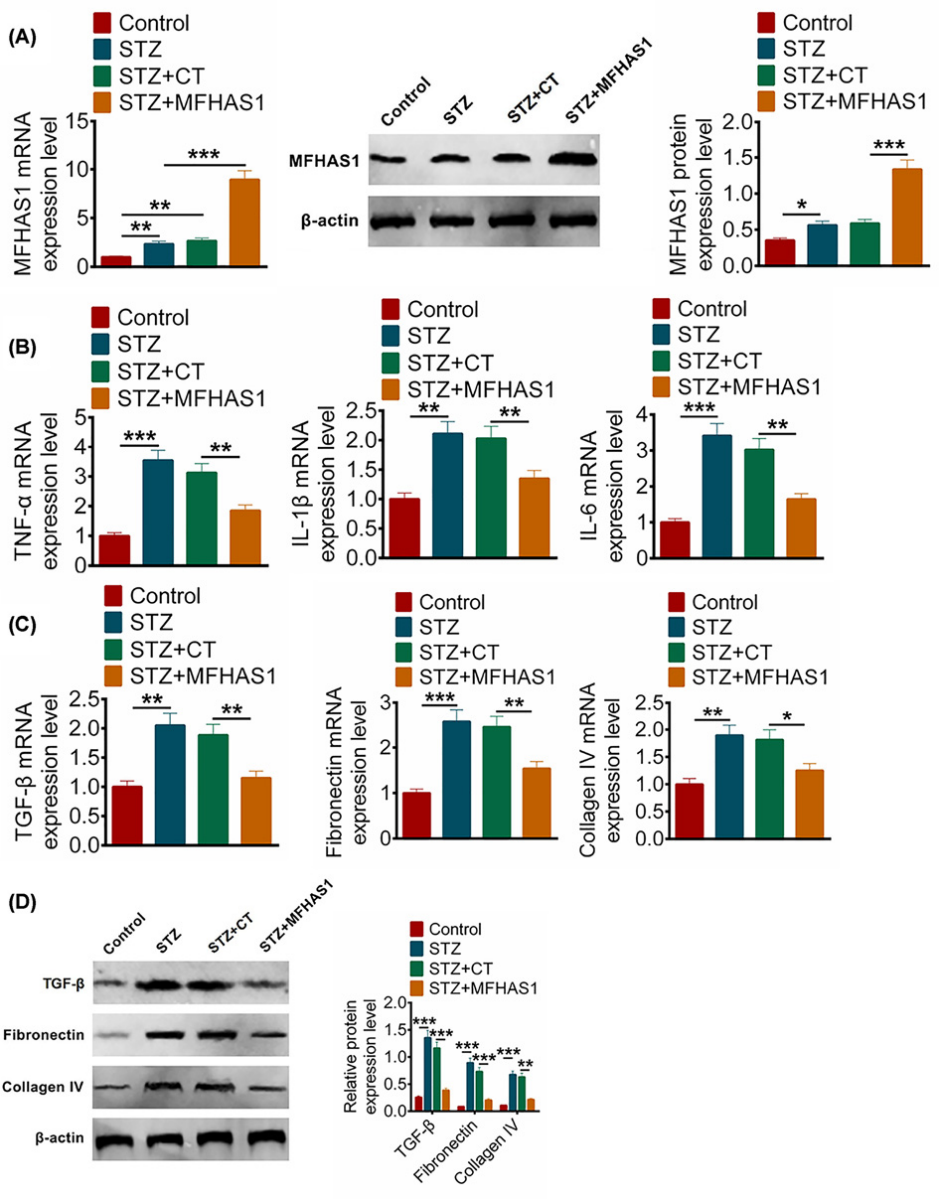
Overexpression of MFHAS1 *in vivo* improved the symptoms of STZ-induced DN mice (**A**) Relative expression levels of MFHAS1 in STZ mice tissues after transfection with CT or MFHAS1 overexpression plasmid and normal tissues were identified using qRT-PCR and Western blot. (**B**) TNF-α, IL-1β and IL-6 expression levels were detected using qRT-PCR after transfection with CT or MFHAS1 overexpression plasmid. (**C**) TGF-β, fibronectin and collagen IV expression levels were detected using qRT-PCR after transfection with CT or MFHAS1 overexpression plasmid. (**D**) TGF-β, fibronectin and collagen IV expression levels were detected using Western blot after transfection with CT or MFHAS1 overexpression plasmid. Data were shown as mean ± SD. *P<0.05, **P<0.01, ***P<0.001 compared with control or STZ + CT group.

To explore the effect of MFHAS1 on DN *in vivo*, STZ mice models were constructed and then CT or MFHAS1 overexpression plasmid was injected into STZ mice via the tail vein. Mice were divided into four groups (control, STZ, STZ + CT, STZ + MFHAS1), six mice per group. To explore the biological role of MFHAS1 in DN mice, the body weight, kidney weight, and physiological indicators of renal function were determined. As observed in Table 1, body weight showed no significant difference in the four groups. Compared with control group mice, kidney weight was increased (*P*<0.05), blood glucose, triglyceride and free fatty acids (FFA) were dramatically increased (*P*<0.01) by STZ treatment. There was no statistical significance of creatinine in the four groups. Twenty-four hour urinary albumin excretion (UAE) was significantly increased in the STZ and STZ + CT groups compared with the control mice, which was decreased in STZ + MFHAS1 mice compared with STZ + CT group (*P*<0.05). After 12-week injection, the mice were put to death and then kidneys were collected and harvested for further researching. The expression levels of MFHAS1, inflammatory factors and fibrosis-related factors were measured via qRT-PCR and Western blot. MFHAS1 expression was markedly enhanced in STZ + MFHAS1 group compared with other groups (*P*<0.001) (Figure 5A). It illustrated that MFHAS1 was availably overexpressed by MFHAS1 overexpression plasmid injection *in vivo*. Figure 5B showed MFHAS1 overexpression decreased the expression levels of TNF-α, IL-1β or IL-6. It indicated that MFHAS1 could inhibited inflammation *in vivo*. In addition, TGF-β, fibronectin and collagen IV expressions were obviously down-regulated in STZ+MFHAS1 group when compared with other groups (*P<*0.001) (Figure 5C,D) in STZ mice. These results demonstrated that overexpression of MFHAS1 effectively relieved the symptoms of DN *in vivo* experiment.

**Table 1:** Physical and biochemical parameters of experimental animals

	Control	STZ	STZ+CT	STZ+MFHAS1
Body weight (g)	26.24 ± 2.96	26.11 ± 2.74	27.41 ± 2.06	29.12 ± 3.09
Kidney weight (g)	0.14 ± 0.01	0.26 ± 0.02^1^	0.28 ± 0.03^1^	0.27 ± 0.03^1^
Blood glucose (mmol/l)	6.33 ± 0.98	19.37 ± 1.71^2^	21.25 ± 1.81^2^	22.56 ± 1.74^2^
Triglyceride (mg/dl)	142.81 ± 12.41	278.28 ± 19.74^2^	284.27 ± 21.18^2^	272.22 ± 19.58^2^
FFA (mmol/l)	1.57 ± 0.13	2.89 ± 0.84^2^	2.77 ± 0.61^2^	2.71 ± 0.65^2^
Creatinine (μmol/l)	62.57 ± 6.87	68.11 ± 8.21	67.52 ± 6.74	63.14 ± 7.12
UAE (μg/24 h)	35.25 ± 2.85	175.22 ± 10.56^2^	184.20 ± 15.24^2^	145.29 ± 12.34^2,3^

Values are means ± SD.

^1^*P*<0.05. ^2^*P*<0.01 vs. Control group. ^3^*P*<0.05 vs. STZ+CT group.

## Discussion

DM is a global disease with a rapid growth rate. Approximately one-third of DM patients develop into DN, which is one of the main causes of disability and death in diabetic patients, and also the main cause of end-stage renal disease. Early DN is characterized by persistent microalbuminuria, progressive progress to high levels of albuminuria and serum creatinine, and ultimately renal failure, which requires dialysis and kidney transplantation. Molecular approaches have been widely used in the treatment of DN. Hong et al. [23] reported that lincRNA-Gm4419 knockdown could improve inflammation mediated by NF-κB/NLRP3 (NOD-like receptor family, pyrin domain containing 3) inflammasome in DN. Shi et al. [24] found that the overexpression of sirtuin 4 (SIRT4) protected against DN via inhibiting podocyte apoptosis. Xu et al. [25] reported that epidermal growth factor receptor (EGFR) knockdown relieved DN through decreasing endoplasmic reticulum stress and reactive oxygen species (ROS). However, the occurrence and progression of DN can not be completely prevented. Therefore, it is particularly important to further study the mechanism of DN and seek new therapeutic strategies and targets.

MFHAS1 is a member of ROCO protein family, and it is associated with innate immunity [16,26]. In previous studies, MFHAS1 was mostly reported as a predictor of malignant fibrous histiocytoma and gastrointestinal tumors [18,19]. Besides, Zhong et al. [27] reported that MFHAS1 was connected with sepsis and stimulated TLR2/NF-κB pathway. MFHAS1 was reported to inhibit HG-induced inflammation by activating AKT/HO-1 pathway in DM [20]. In the current study, we first studied the effect of MFHAS1 on DN.

Db/Db mice and STZ mice are common animal models that can be used in DN research [28,29]. In the present study, we examined the expression level of genes in DN tissues through db/db mice and STZ mice, and then found that MFHAS1 expression was significantly up-regulated in DN tissues and cells compared with normal tissues and cells (*P*<0.001). The overexpression and downexpression plasmids of MFHAS1 were subsequently constructed and then transfected into MMCs with LG or HG treatment. Our study indicated that HG treatment obviously promoted MFHAS1 expression when compared with LG treatment (*P*<0.001). And the MFHAS1 overexpression inhibited the expression levels of inflammatory factors (TNF-α, IL-1β, IL-6) and fibrosis-related genes (TGF-β, fibronectin, collagen IV).

In the current study, MFHAS1 was detected to be up-regulated in DN mice tissues and cells and showed a renoprotective effect in the diabetic mice. We suspected that this may be due to the action of MFHAS1 on TRL4. It was reported that MFHAS1 could act on the expression of TLR4 in previous study, and the overexpression of MFHAS1 could inhibit the TLR4 signaling pathway [21]. TLR4 expression was up-regulated in tissues and cells of DN mice, and we hypothesized that TLR4 overexpression induced MFHAS1 overexpression. To verify our prediction, the expression level of TLR4 after transfecting shNC or shRNA were detected via qRT-PCR and Western blot. We found that TLR4 expression decreased while MFHAS1 expression increased. In addition, short hairpin TLR4 (shTLR4) or TAK242, that could knockdown TLR4, were co-transfected with shMFHAS1/shN to verify the predication. Our results showed that the inflammation and fibrosis were both suppressed when the TLR4 was down-regulated. It exhibited that MFHAS1 inhibited inflammation and fibrosis through suppressing TLR4 expression. And the specific mechanism of MFHAS1 effecting on TLR4 in DN might be a key point in the future.

Besides, we examined the effect of MFHAS1 on DN *in vivo* via injecting CT or MFHAS1 overexpressed plasmid into STZ mice. The MFHAS1 expression of STZ + MFHAS1 group was significantly higher than other groups. It illustrated that FHAS1 was successfully overexpressed in STZ mice after injection. TNF-α, IL-1β and IL-6, inflammatory factors, were all inhibited in STZ mice with MFHAS1 injection. Similarly, the expressions of TGF-β, fibronectin and collagen IV were also suppressed after MGHAS1 injection. These results demonstrated that MFHAS1 could effect DN *in vivo*.

In conclusion, the data of the present study indicated that MFHAS1 played a negative role in DN inflammation and fibrosis. Overexpression of MFHAS1 inhibited inflammation and fibrosis both *in vivo* and *in vitro* through suppressing the activation of TLR4. The current results revealed that MFHAS1 may be a molecular therapeutic target in DN.

## Availability of Data and Materials

All data generated and/or analyzed during the present study are included in this published article.

## Abbreviations

DM: diabetes mellitus
DN: diabetic nephropathy
EGFR: epidermal growth factor receptor
ESRD: end-stage renal dialysis
HG: high glucose
LG: low glucose
LPS: lipopolysaccharide
MFHAS1: malignant fibrous histiocytoma amplified sequence 1
MMC: mouse mesangial cell
NLRP3: NOD-like receptor family, pyrin domain containing 3
PP2A: protein phosphatase 2A
shNC: short hairpin negative control
shRNA: short hairpin RNA
STZ: streptozotocin
TBS-T: Tris-buffered saline containing 0.1% Tween-20
TLR4: Toll-like receptor 4
ROCO: C-terminal of Ras of complex proteins
ROS: reactive oxygen species
SIRT4: sirtuin 4
